# Developmental improvements in the ability to benefit from testing across middle childhood

**DOI:** 10.3389/fnbeh.2024.1501866

**Published:** 2024-12-18

**Authors:** Sandra Rodríguez-Gonzalo, Jaione Arnaez-Telleria, Pedro M. Paz-Alonso

**Affiliations:** ^1^Basque Center on Cognition, Brain and Language (BCBL), Donostia-San Sebastián, Spain; ^2^IKERBASQUE, Basque Foundation for Science, Bilbao, Spain

**Keywords:** testing effect, retrieval practice, long-term memory, middle-childhood, early adolescence

## Abstract

Extensive behavioral research on adults has shown that retrieval practice is highly beneficial for long-term memory retention. However, limited evidence exists on the developmental course of this benefit. Here, we present data from a behavioral study involving 7–14-year-old children who had to encode a total of 60 weakly semantically related cue-target word pairs using either repeated retrieval or repeated study encoding strategies. Results revealed age-related increases in the ability to benefit from testing during encoding from early middle childhood to early adolescence. In contrast, repeated study during encoding did not lead to developmental improvements in long-term memory retention across this age range. Individual differences in vocabulary knowledge, short-term memory and working memory were positively associated with long-term memory retention only for those participants who encoded the information via repeated study. These results indicate that (1) the mechanisms determining the testing effect may not be fully in place by early middle childhood, (2) the ability to benefit from testing improves over the middle childhood years, and (3) these benefits are not associated with individual differences in memory and high-cognitive functioning. One potential interpretation of these findings is that changes in sleep-dependent consolidation processes during middle childhood may be critical for understanding the observed developmental differences in ability to enhance long-term memory via the testing effect.

## 1 Introduction

In modern societies, individuals are constantly exposed to new to-be-learned information and skills, such as acquiring a new language (Barcroft, 2007; Karpicke and Bauernschmidt, 2011), learning motor sequences (Tempel and Frings, 2019), and learning curricular contents and procedures in educational settings (McConnell et al., 2015). Identifying mnemonic strategies that allow for efficient information encoding and lasting long-term memory retention hold much promise to advance human memory theory and research, and it could have important translational benefits in educational and clinical settings.

Over the last few years, there has been increased interest in examining the beneficial effects of testing on long-term memory. Extensive behavioral evidence from adults has shown the robustness of the so-called testing effect (Karpicke and Roediger, 2008; Roediger and Karpicke, 2006a) using a wide range of materials (e.g., Butler and Roediger, 2007; Carpenter and DeLosh, 2006; Kliegl and Bäuml, 2021; McDaniel et al., 2007; Pyc and Rawson, 2010; Schuetze et al., 2019), types of memory tests (e.g., Eglington and Kang, 2018; Karpicke and Zaromb, 2010; Ludowicy et al., 2023; Odegard and Koen, 2007; Roediger and Marsh, 2005) and retention intervals (e.g., Roediger and Karpicke, 2006a, 2006b; Toppino and Cohen, 2009; Wheeler et al., 2003). The testing effect (i.e., benefits of retrieval practice) refers to an increased strength of memory related to some information after actively retrieving (e.g., through cued recall) as opposed to re-studying that information (Roediger and Karpicke, 2006b). Since retrieving information after learning it reinforces the memory of that information, the term “backward testing effect” is also frequently used to describe the same phenomenon. Indeed, while the effect sizes of the testing effect are considered relatively large (see Adesope et al., 2017 for a meta-analytic review), recall tests as opposed to recognition tests, and 1-day retention intervals as opposed to shorter retention intervals (e.g., minutes or hours) yield even larger testing effect benefits (Rowland, 2014). These findings are consistent with theoretical accounts emphasizing effortful processing as a contributor to the testing effect (e.g., Bjork and Bjork, 1992; Glover, 1989; Pyc and Rawson, 2009).

Importantly, the testing effect has also been demonstrated in a number of studies conducted with children at different developmental periods: during preschool years (aged 3–5; Fritz et al., 2007), early middle childhood years (aged 7–9; Aslan and Bäuml, 2015; Bouwmeester and Verkoeijen, 2011; Lipowski et al., 2014), and late middle childhood years (aged 9–10; e.g., Rohrer et al., 2010; see also Fazio and Marsh, 2019, for a developmental review on the testing effect). Studies that have implemented testing procedures during learning in actual classroom contexts suggest that the general memory benefits observed in laboratory research generalize to these real settings and promote learning across secondary school or middle school years (e.g., Carpenter et al., 2009; McDaniel et al., 2011; McDaniel et al., 2013; Roediger et al., 2011), high school or adolescence (e.g., McDermott et al., 2014), and university or young adulthood (Bangert-Drowns et al., 1991; Bjork et al., 2014; Gingerich et al., 2014; Leeming, 2002; McDaniel et al., 2007).

Nevertheless, the developmental course of the testing effect is still unclear. To what extent do age-related improvements in the ability to benefit from the testing effect occur during middle childhood years? Are individual differences in memory performance and higher cognitive function positively associated with long-term memory retention as a function of repeated study or repeated retrieval encoding strategies? These are critical questions for developmental science, which can also provide important information for the implementation of testing-related programs in educational settings and shed light on the underlying mechanisms supporting the testing effect. For instance, a fixed ability to benefit from the testing effect from early middle childhood onwards would suggest that the testing effect is relatively independent of the wide range of long-term memory functions observed to improve over the middle childhood years. In contrast, if age-related improvements in the ability to benefit from multiple testing occurs mainly over middle childhood years, this may suggest that other concomitant neurobiological and mnemonic improvements that also take place during this period influence the testing effect (e.g., Ghetti and Angelini, 2008; Giedd, 2008; Paz-Alonso et al., 2009; Paz-Alonso and Goodman, 2016; Shaw et al., 2008; Tamnes et al., 2010; Tang et al., 2018).

Previous developmental work specifically examining age-related differences in the ability to benefit from testing during childhood and adolescence has primarily focused on the “forward testing effect” (Aslan and Bäuml, 2015; Dang et al., 2022). The forward testing effect - a variation of the more traditional backward testing effect (Pastötter and Bäuml, 2014; Szpunar et al., 2008; Yang et al., 2017), refers to the fact that engaging in testing to encode certain items leads to longer-lasting memories for subsequently encoded new items (see Yang et al., 2018, for a review). Findings from developmental studies using the forward testing effect in children are mixed. Aslan and Bäuml’s (2015) study found a benefit of testing over restudying in early middle childhood (8–9-year-olds) and adult groups, but not in late preschoolers (6–7-year-olds), suggesting that age positively influences the ability to benefit from testing. In contrast, studies by Dang et al. (2022) and Aslan and Kubik (2024) found that the forward testing effect can be observed from preschool years on.

The aim of the present study is 2-fold: (1) to examine the developmental trajectories of the beneficial effects of the backward testing effect from early middle childhood to early adolescence; and (2) to investigate to what extent individual differences in memory and higher cognitive functioning support age-related variations in this ability to benefit from the testing effect. To this end, we used a sample of children aged 7–14-years-old, covering the entire middle childhood and early adolescence, to examine the more traditional “backward testing effect” in which participants typically show an enhancement of memories encoded via repeated retrieval versus repeated study. One of the advantages of the backward testing effect is that it allows control over what information is or is not encoded in long-term memory after the learning phase, and it also ensures that once an association has been encoded, that memory will only and exclusively be reinforced either via repeated study or repeated retrieval. Nevertheless, since the backward testing effect either emphasizes repeated study or retrieval practice but includes both study and retrieval trials in both encoding agendas (Karpicke et al., 2014), it has been pointed out that this paradigm may not only reflect participants’ ability to learn from testing but also participants’ ability to learn from feedback/study, at least until associations are learned. We acknowledge this possibility, especially considering that learning from feedback/study can be also subjected to developmental changes. We also think that it is important to highlight that to go through study and retrieval attempts until information gets encoded is more naturalistic in real life situations than just undergoing pure testing without studying or pure studying without testing. In this sense, a third advantage of the backward testing effect is that it better resembles learning in natural situations.

Despite previous developmental work showing that children at different developmental stages can benefit from testing (Aslan and Bäuml, 2015; Aslan and Kubik, 2024; Bouwmeester and Verkoeijen, 2011; Dang et al., 2022; Fritz et al., 2007; Lipowski et al., 2014), and extensive evidence showing critical improvements in long-term memory-related processes across middle childhood (Ghetti and Angelini, 2008; Ghetti and Bunge, 2012; Paz-Alonso et al., 2013b; Paz-Alonso et al., 2008; Yu et al., 2018), no studies to date have specifically examined age-related changes in the ability to benefit from the classical backward testing effect across middle childhood years. We predicted that testing would confer more benefits during late middle childhood/early adolescence than during early middle childhood. Finally, to better understand to what extent individual differences in memory and higher-cognitive functioning are associated with long-term memory retention, in the present study, we administered a battery of tests to measure cognitive skills, including vocabulary knowledge, fluid reasoning, short-term memory, working memory, speed of processing and memory recognition. We expected that individual differences in memory and higher-cognitive functioning would predict long-term memory in the repeated study group participants, but not in the participants assigned to the repeated retrieval group (Agarwal et al., 2017; Pastötter and Frings, 2019). This result would suggest that memory enhancement produced by the testing effect is mainly associated with the retrieval practice itself rather than individual differences in these measurements.

## 2 Methods

### 2.1 Participants

Eighty-one Spanish-speaking children were recruited and took part in the study. All subjects had normal or corrected-to-normal vision and no history of learning disabilities. Data from 4 participants were excluded due to not being able to learn at least 75% of the to-be-studied word pairs or having outlier values (mean ± 2.5 * standard deviation) in the final test administered 2 days after encoding, leaving a final sample of 77 subjects (M = 11.03 years; SD = 2.12 years; 41 females). The sample was divided into four groups based on the corresponding age group and learning procedure. The age division was made according to the critical improvements observed half way through middle childhood years (e.g., Ghetti and Angelini, 2008; Paz-Alonso et al., 2009; Paz-Alonso et al., 2014), resulting in a Younger children group (i.e., middle-childhood, ages 7–10) and an Older children group (i.e., early-adolescence, ages 11–14). The second division, based on the initial procedure to commit items to memory, separated the participants into Repeated Study group (i.e., learning mainly through re-study) or Repeated Retrieval group (i.e., learning mainly through testing). According to this criterion, our final sample consisted of younger participants (i.e., between 7 and 10 years old) who encoded the items either under repeated study conditions (Younger Repeated Study group: *n* = 18, M = 9.21 years; SD = 1.16 years; 10 females), or via repeated retrieval (Younger Repeated Retrieval group: *n* = 20, M = 9.20 years; SD = 1.07 years; 10 females); and older participants (i.e., between 11 and 14 years old) who encoded the items engaging either in repeated study (Older Repeated Study group: *n* = 20, M = 12.81 years; SD = 1.07 years; 9 females) or repeated retrieval (Older Repeated Retrieval group: *n* = 19, M = 12.82 years; SD = 1.19 years; 12 females). All participants were recruited from the same school in Vitoria (Spain) at which Spanish, Basque and English are taught from preschool to school years with a similar degree of exposure to these languages across these years. Prior to taking part in the experiment, participants’ parents gave written informed consent in compliance with the ethical regulations established by the Ethics Committee of our research center and the guidelines of the Helsinki Declaration.

Within each age group, repeated study and repeated retrieval encoding groups resulted to be matched in gender and individual difference variables, including: vocabulary knowledge and fluid reasoning (Kaufman Brief Intelligence Test, K-BIT-II; Kaufman and Kaufman, 1990), working memory (Woodcock-Johnson-III, numbers reversed and auditory working memory subtests; Woodcock et al., 2001), speed of processing (Woodcock-Johnson-III, cross-out subtest; Woodcock et al., 2001), short-term visual memory (Cambridge Neuropsychological Test Automated Battery, CANTAB; Delayed Matching to Sample, DMS, and Pattern recognition memory, PRM subtests) and visual memory recognition (CANTAB; Paired Associates Learning, PAL) (see Table 1).

**Table 1:** Age and individual difference comparisons between repeated study and repeated retrieval encoding groups within each age group.

Younger	Repeated study	Repeated retrieval	*p*-values
Age	9.21 (±1.16)	9.20 (±1.07)	0.98
Vocabulary knowledge	47.89 (±9.53)	47.45 (±8.54)	0.88
Fluid reasoning	29.61 (±5.20)	29.40 (±4.25)	0.89
Working memory	502.61 (±18.20)	506.05 (±14.63)	0.53
Speed of processing	500.28 (±13.01)	495.90 (±10.44)	0.26
Delayed matching to sample	7.83 (±1.92)	7.00 (±1.83)	0.18
Paired associates learning	6.39 (±1.09)	6.20 (±1.36)	0.64
Pattern recognition memory	20.39 (±2.89)	19.35 (±2.52)	0.25

Older	Repeated study	Repeated retrieval	*p*-values
Age	12.81 (±1.07)	12.82 (±1.19)	0.96
Vocabulary knowledge	57.75 (±6.79)	60.58 (±4.23)	0.13
Fluid reasoning	35.25 (±4.96)	36.84 (±4.02)	0.28
Working memory	522.70 (±20.86)	522.94 (±16.25)	0.97
Speed of processing	519.00 (±13.46)	519.68 (±20.88)	0.90
Delayed matching to sample	8.70 (±1.75)	8.95 (±1.71)	0.65
Paired associates learning	6.55 (±0.94)	6.37 (±0.95)	0.55
Pattern recognition memory	21.45 (±2.46)	21.00 (±1.73)	0.51

Standard deviation in parentheses.

### 2.2 Stimuli

A total of 60 Spanish weakly semantically related cue-target word pairs (e.g., hole-cheese) were used. Thirty new cue words were also selected for the retrieval test that took place 2 days after the encoding happened to ensure that participants were engaging in the task by reducing the frustration they could experience when not remembering studied items. Participants exhibited a near-perfect accuracy at withholding responses to these non-studied novel cues. All word stimuli were classified as words typically acquired by age 7. Moreover, cue words, target words and new cue words were matched for age of acquisition, familiarity and imageability psycholinguistic norms (*p*s ≥ 0.16).

### 2.3 Procedure

Participants from both learning groups (i.e., repeated study, repeated retrieval) underwent two experimental sessions 2 days apart from each other. They were randomly assigned to the repeated study and repeated retrieval conditions. In session I (i.e., initial), participants were presented with the 60 Spanish word pairs to commit to memory. Session I was divided into 8 study-test cycles wherein participants studied the to-be-remembered word pairs under either the repeated study or repeated retrieval learning conditions depending on their assigned group. Each cycle consisted of a study phase, where the word pair (e.g., hole - cheese) appeared on the computer screen for 7,000 ms while they attempted to memorize it, and a cued recall test phase, where participants were shown the first word of the word pair for 5,700 ms (e.g., hole - ____) and had to verbally recall the second word of the pair.

Younger and older participants in the repeated study groups studied all of the 60 experimental word pairs in each of the 8 study-test cycles and were only re-tested on those items that they did not remember in the immediately preceding cycle. Younger and older participants in the repeated retrieval groups retrieved the 60 experimental word pairs in all of the 8 study-test cycles. They were only asked to re-study those items they were unable to remember in the immediately preceding cycle. Thus, while both groups underwent a study and a retrieval phase for them to learn the pairs and assess whether they had learned them correctly, each group focused on either re-studying or re-testing to commit the pairs to memory. Participants who were not able to correctly remember at least 45 word pairs by the end of the 8th study-test cycle were excluded (*n* = 3; the remaining excluded participant exhibited an outlier performance in the final test, 2 days after encoding). The low number of participants who were not able to successfully encode at least 75% of the word pairs indicates that the majority of the children were able to understand and perform the task.

Table 2 shows the encoding procedure in 8 study-test cycles and the average number of stimuli presented in each cycle, as a function of Age (younger children, older children) and Encoding group (repeated study, repeated retrieval). Importantly, the overall amount of exposure to the stimuli did not vary significantly between the repeated study and repeated retrieval conditions, either in the older children group (*p* = 0.80) or in the younger children group (*p* = 0.08). Nevertheless, to rule out the possibility that individual differences in the degree of exposition to the word pairs during encoding may determine developmental effects, we tested whether or not controlling the number of expositions in our main analysis had an influence on the results.

**Table 2:** Behavioral encoding procedure and average number of studied/tested trials in each of the 8 study-test cycles as a function of age group and encoding group.

Younger	1		2		3		4		5		6		7		8		Total number of trials
	S	T	S	T	S	T	S	T	S	T	S	T	S	T	S	T	
Repeated study	60	60	60	33.8	60	18.2	60	8.5	60	4.2	60	2.2	60	1.1	60	0	608.0 (48.7)
Repeated retrieval	60	60	35.9	60	22.1	60	13.1	60	7.2	60	4.1	60	2.3	60	1.2	60	625.9 (50.9)

Older	1		2		3		4		5		6		7		8		Total number of trials
	S	T	S	T	S	T	S	T	S	T	S	T	S	T	S	T	
Repeated study	60	60	60	31.6	60	16.1	60	9	60	4.8	60	2.3	60	0.9	60	0.2	604.9 (47.5)
Repeated retrieval	60	60	30.3	60	15.5	60	7.8	60	4.0	60	2.2	60	1.5	60	0.7	60	602.0 (49.8)

Standard deviation in parentheses. S, study; T, test.

In session II, which took place 2 days after session I, participants performed a final cued recall test. Participants were presented with the cue words for the previously studied 60 word pairs (e.g., hole -___) and were asked to respond aloud (e.g., “cheese”) while the cue was on the screen (i.e., 5,700 ms). Together with the studied cue words, participants encountered 30 new Spanish cue words that had not previously been presented to the participants during the encoding phase to reduce the frustration they could experience when not remembering studied items. They were encouraged to say “pass” anytime they did not know the response to a given cue word.

## 3 Results

**Figure 1:**
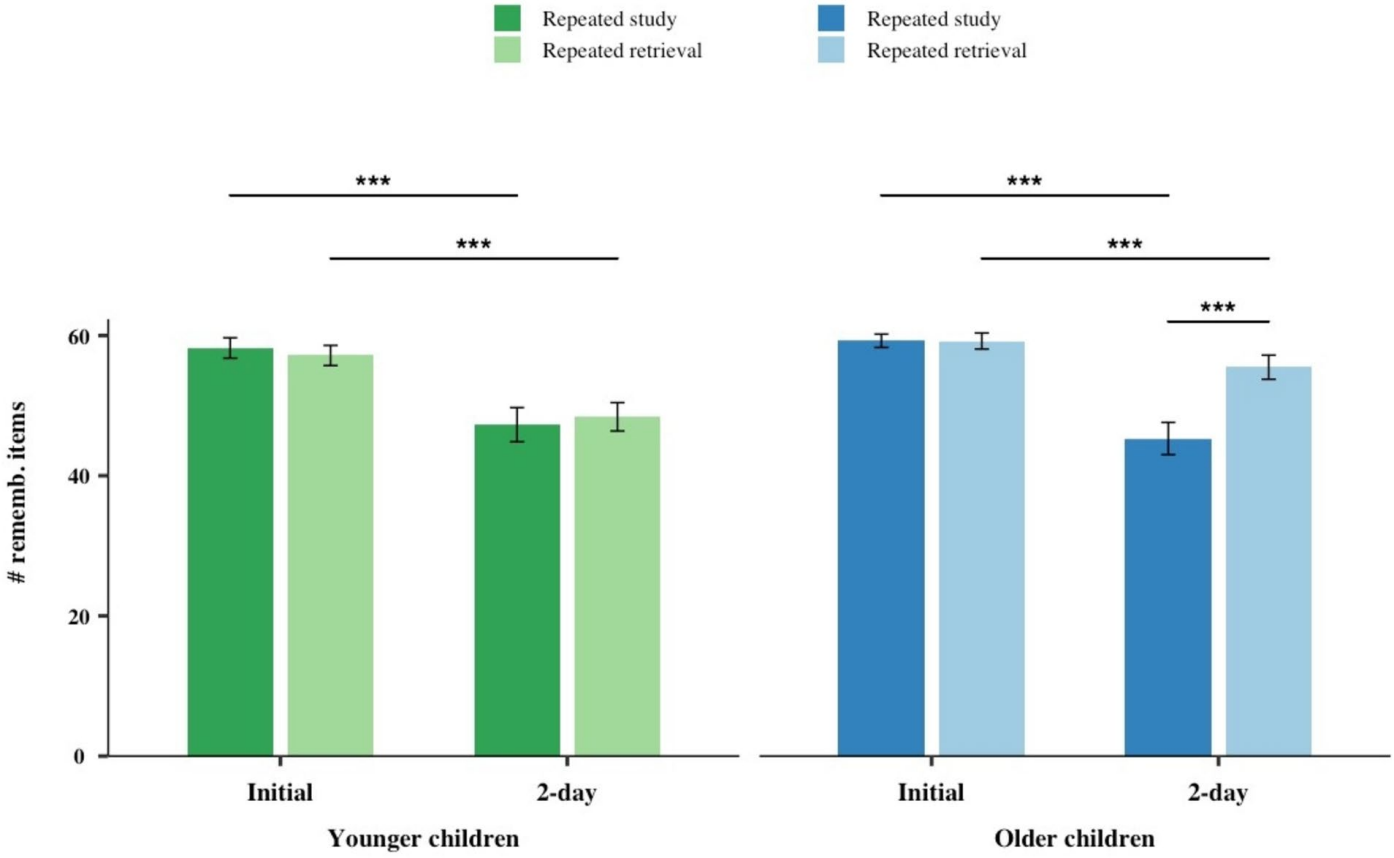
Number of correctly remembered items as a function of Age group (younger children, older children), Session (initial, 2-day) and encoding group (repeated study, repeated retrieval). The error bars represent the standard error. *p < 0.05, **p < 0.01, ***p < 0.001. rememb., remembered.

A mixed-model ANOVA including all the experimental factors: 2 (Age group: younger children, older children) × 2 (Encoding group: repeated study, repeated retrieval) × 2 (Session: initial, 2-day), with the latter factor varied within participants, was conducted with the number of correctly remembered items as the dependent measure. For the initial session, we used the number of remembered items at the end of the encoding session (i.e., final number of word-pairs they were able to commit to memory after the 8 cycles) and for the 2-day session, we used the number of correctly retrieved items at the final test. This analysis revealed that the statistically significant main effect of Session [*F*_(1, 73)_ = 175.66, *p* < 0.001, $\eta_{p}^{2}$ = 0.71] was qualified by an Encoding group × Session interaction [*F*_(1, 73)_ = 19.35, *p* < 0.001, $\eta_{p}^{2}$ = 0.21] and an Age group × Encoding group × Session second-order interaction [*F*_(1, 73)_ = 8.08, *p* < 0.01, $\eta_{p}^{2}$ = 0.10] (see Figure 1).

Given that it has been pointed out that the backward testing effect may rely to some extent on participants’ ability to learn from feedback/study, we conducted the same analysis controlling for the number of expositions to the to-be-encoded words during encoding. The results of this ANCOVA showed that the same effects resulted statistically significant, including the Encoding group × Session interaction [*F*_(1, 72)_ = 27.11, *p* < 0.001, $\eta_{p}^{2}$ = 0.27] and the Age group × Encoding group Session second-order interaction [*F*_(1, 72)_ = 5.29, *p* < 0.05, $\eta_{p}^{2}$ = 0.07].

To examine the higher order interaction, simple effect post-hoc analyses were conducted separately for the younger and older children groups. In both age groups, there was a statistically significant decrease in the number of correctly remembered items from the initial session to the 2-day session across both the repeated study and repeated retrieval conditions [*t*s(17–19) ≥ 4.54, *p*s ≤ 0.001, *d*s > 1.04]. Also, in both age groups no statistically significant differences emerged between encoding groups in terms of the number of correctly remembered items at the initial session [*t*s(36–37) ≤ 0.99, *p*s ≥ 0.33, *d*s ≥ 0.022]. In contrast, a post-hoc analysis revealed that this interaction was due to the fact that the older group showed a statistically significant difference between repeated retrieval versus repeated study conditions at the 2-day session [*t*(37) = 4.22, *p* < 0.001, *d* = 1.38], an effect that was not observed in the younger children group [*t*(36) = 0.44, *p* = 0.66, *d* = 0.15]. To further test that there was a significant difference between the repeated retrieval versus repeated study in the number of correctly recalled items in the 2-day session, we carried out a 2 (Age group: younger children, older children) × 2 (Encoding group: repeated study, repeated retrieval) ANOVA with the performance only in the 2-day session. This analysis revealed a statistically significant Age group × Encoding group interaction [*F*_(1, 73)_ = 6.74; *p* = 0.011; $\eta_{p}^{2}$ = 0.09].

**Figure 2:**
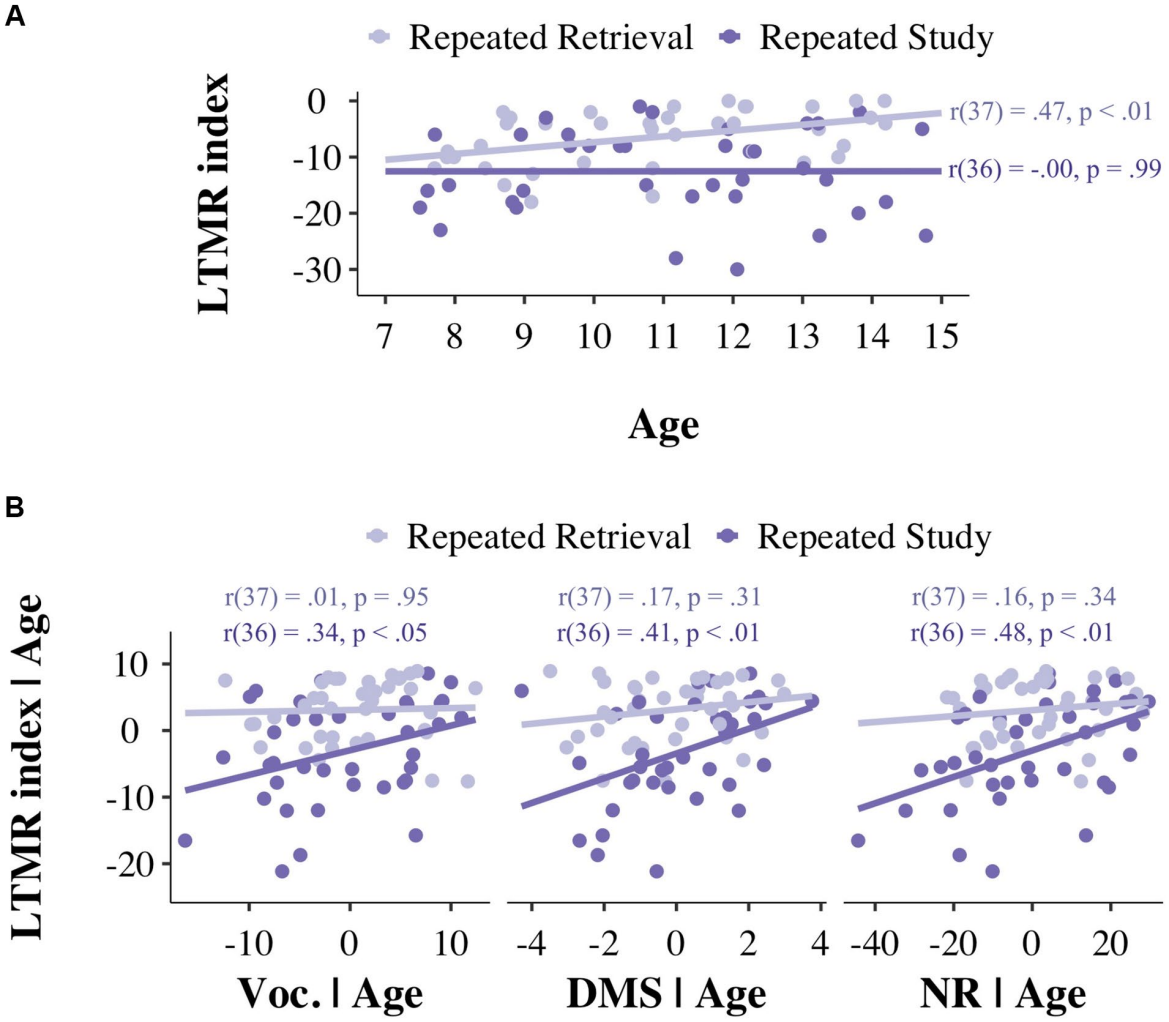
**(A)** Scatterplot of the associations between the long-term memory retention index and Age as a function of Encoding group: repeated study (dark purple) and repeated retrieval (light purple). **(B)** Scatterplots showing age corrected linear associations between the long-term memory retention index and individual differences in semantic knowledge, short-term memory and working memory, as a function of encoding group. LTMR, Long-term memory retention; Voc., Vocabulary; DMS, Delayed matched to sample; NR, Numbers reversed.

Since the testing effect can also be defined as the difference in the number of forgotten items from session I to session II between the repeated study and repeated retrieval groups, we created a dependent measure that represents this difference: the long-term memory retention index, defined as the number of correctly recalled items at the 2-day session minus the number of items that were able to correctly encode in the first session. Due to the way we defined this variable, larger negative numbers represent more forgotten items. To further examine to what extent age was associated with children’s ability to benefit from testing, we conducted simple regression analyses between age and the long-term memory retention (LTMR) index separately for the repeated retrieval and the repeated study encoding groups (see Figure 2A). For the repeated retrieval group, there was a statistically significant association between age and the LTMR index [*r*(37) = 0.47, *p* < 0.01]. In contrast, there was no significant association between age and the LTMR index in the repeated study group [*r*(36) = −0.00, *p* = 0.99], and neither was there a significant difference in the LTMR-age association in the repeated retrieval group versus the repeated study group [*t*(73) = −1.59, *p* = 0.12].

Another goal of the present study was to examine if individual differences in memory and higher-cognitive functioning predicted participants’ performance as a function of the encoding group. To this end, we carried out a series of age-corrected regression analyses between the LTMR index and raw scores from individual difference tests measuring higher cognitive functions (i.e., K-BIT-II, working memory, cross-out) and memory functioning (CANTAB subtests: DMS, PRM, PAL).

Individual differences in the raw scores for vocabulary knowledge (K-BIT-II), short-term memory (CANTAB, DMS subtest) and working memory (Woodcock-Johnson-III numbers reversed subtest, NR) were positively associated with the LTMR index in the repeated study group [K-BIT-II: *r*(36) = 0.34, *p* < 0.05; DMS: *r*(36) = 0.41, *p* < 0.01; NR: *r*(36) = 0.48, *p* < 0.01]. In contrast, there were no statistically significant associations of the LTMR index with K-BIT-II, DMS or NR in the repeated retrieval group [K-BIT: *r*(37) = 0.01, *p* = 0.95; DMS: *r*(37) = 0.17, *p* = 0.31; NR: *r*(37) = 0.16, *p* = 0.34] (see Figure 2B), and the differences in these associations between the repeated study group versus the repeated retrieval group were not statistically significant [*t*s(73) ≤ 1.77, *p* ≥ 0.08]. Since the LTMR index of the repeated study group did not show significant associations with age, we also performed the same simple regression analyses for this group without correcting for age. As in the age corrected correlations, the repeated study group’s LTMR index was positively associated with vocabulary knowledge, short-term memory and working memory scores [K-BIT: *r*(38) = 0.31, *p* < 0.05; DMS: *r*(38) = 0.38, *p* < 0.01; NR: *r*(38) = 0.42, *p* < 0.01].

## 4 Discussion

The present study was aimed at investigating age-related changes in the ability to benefit from the backward testing effect over the middle childhood years, as well as to examine to what extent individual differences in memory and high-cognitive functioning variables may support potential developmental variations in long-term memory retention. Our findings indicate that (1) the memory gains produced by testing are subjected to developmental changes over the middle childhood years; and (2) long-term memory retention is associated with individual differences in vocabulary knowledge, short-term memory and working memory in the repeated study, but not in the repeated retrieval group. These results are discussed next.

### 4.1 Developmental changes on the testing effect

The paradigm we utilized in the present study has been extensively used in the testing effect literature. The backward testing effect paradigm is of special interest in controlling for the items encoded in long-term memory by the end of the learning phase, which is critical in order to level the playing field among developmental groups in this sense. It is also of special interest in making sure that once a memory is encoded, that memory is only reinforced either through repeated retrieval or repeated study, as a function of the encoding group conditions. Across several analyses, our results demonstrate that the effects of testing differ across the middle childhood years. All groups were able to encode most of the to-be-encoded items and performed at ceiling in the first session, but in the second session (2 days after encoding), only older children exhibited a testing benefit. In fact, the only group that differed in the number of correctly retrieved items 2 days after encoding was the older repeated retrieval group. Importantly, this developmental effect holds when controlling for the ability to learn from feedback/study or the degree of exposition to the to-be-encoded word pairs during the learning phase.

Moreover, the group of participants who engaged in retrieval practice during encoding exhibited a strong association between long-term memory retention and age. This suggests improvements in the ability to benefit from testing from early middle childhood to early adolescence. In contrast, the group of participants who engaged in study practice during encoding did not show statistically significant associations between long-term memory retention and age. These age-related changes in the ability to benefit from the testing effect over the middle childhood years appear to be concomitant with well-documented improvements in other cognitive processes that also occur from early to late middle childhood such as binding operations (Ghetti and Angelini, 2008; Lloyd et al., 2009; Sluzenski and Newcombe, 2006), mnemonic control (Ashcraft et al., 1976; Bunge et al., 2002; Emmerich and Ackerman, 1978; Paz-Alonso et al., 2013a; Tang et al., 2018; Yu et al., 2018) and the ability to process semantically related information (Cunningham and Stanovich, 1991; Paz-Alonso et al., 2013b; Paz-Alonso et al., 2008). Developmental cognitive neuroscience research on memory retrieval have also characterized age-related changes in hippocampo-cortical functional coupling from early middle childhood to adolescence (e.g., Ofen et al., 2012; Paz-Alonso et al., 2013a; Paz-Alonso et al., 2013b) that can be at the base of the developmental differences here observed in the ability to benefit from testing effect.

### 4.2 Individual differences and the testing effect

Besides investigating age-related effects on the testing effect, we also sought to examine the role of individual differences in this phenomenon. Our results revealed that the long-term memory retention of participants who encoded information via repeated study was positively associated with their vocabulary knowledge, short-term memory and their ability to retain and manipulate information in working memory. However, long-term memory retention in the repeated retrieval group did not show any associations with these or other cognitive measures. This finding suggests that variables related to the above-mentioned concomitant changes observed during middle childhood years are not specifically related to the benefits of the testing effect. Only information learnt via the repeated study encoding procedure was associated with other individual difference measures. These results are in line with previous evidence showing that individual differences in working memory and fluid intelligence are associated with the ability to benefit from different encoding agendas (Agarwal et al., 2017; Brewer and Unsworth, 2012). For instance, Agarwal et al.’s (2017) study found that differences in long-term memory retention for information encoded via testing versus repeated study was greater for those participants with lower working memory capacity.

The fact that these associations were only present in the repeated study group suggests that only information encoded via the repeated study condition benefits from these individual difference variables. Differing from repeated testing, the repeated study encoding condition did not include specific instructions regarding the cognitive operations to be performed during encoding of the to-be-remembered items. In this context, previous semantic knowledge may be critical for strengthening or forming new semantic relations between weakly semantically related word-pairs. Similarly, holding information in mind at the short-term memory level and performing operations with this information may be strongly beneficial in terms of binding elements in a word pair together or binding a word pair to other external elements during encoding. This could facilitate subsequent retrieval of the information. It has further been shown that these types of encoding strategies are associated with better long-term episodic memory (see Richardson, 1998 for a review). Thus, under the repeated study condition, encoding strategies may arise spontaneously, depending on individual differences in semantic knowledge and short- and working-memory capacity, leading to better long-term memory retention.

In contrast, the repeated retrieval encoding condition capitalizes on actively performing retrieval operations in order to commit the to-be-remembered information to long-term memory. In this sense, participants exercising retrieval practice have already been provided with specific instructions and strategies. They know what cognitive operations they should engage in during encoding. This suggests, in line with the wealth of research evidence demonstrating the robustness of the testing effect, that testing may *per se* benefit long-term memory retention, without the need for additional encoding strategies.

### 4.3 Concomitant developmental cognitive changes across middle childhood

We confirmed our hypothesis finding age-related improvements in the ability to benefit from the testing effect over the middle childhood years. However, we found these specific testing effects were not related to individual differences in other mnemonic and higher-cognitive functions known to improve over this same developmental period, such as binding operations, relational semantic processing and mnemonic control.

This lack of overlap with concomitant developments in cognitive abilities during middle childhood may be due to the fact that the testing effect differs considerably from other episodic long-term memory paradigms typically used in studies which show improvements in the above-mentioned mnemonic and higher-cognitive functions over the middle childhood years. If so, the testing effect might be governed by different mechanisms. The benefits of testing are typically more robust after a retention interval of at least 24 h (i.e., 2 days in our experiment). These retention intervals, longer than those typically used in paradigms, such as the Desee/Roediger-McDermott (DRM) task (Deese, 1959; Roediger and McDermott, 1995), dual memory paradigms to measure recollection and familiarity (Yonelinas, 1994), the Think/No-Think paradigm (Anderson and Green, 2001), etc., introduce a pivotal new process that also develops over the middle childhood years: memory consolidation. Different studies have shown developmental changes in the sleep-dependent consolidation processes that affect memory consolidation in children from middle childhood through to adolescence. For example, children do not benefit from post-learning periods of sleep in finger sequence tapping and mirror tasks (Prehn-Kristensen et al., 2009; Wilhelm et al., 2008) as adults do (Plihal and Born, 1997; Walker et al., 2003). In fact, using a longitudinal design, Hahn et al.’s (2019) study showed that changes in the distribution of fast spindles toward an adult-like topography (i.e., increased central fast sleep spindle density) occurred between late-middle childhood and adolescence; and that this change was associated with memory consolidation (Hahn et al., 2019).

Although this is just offered as a potential explanation for the age-related changes observed in the ability to benefit from the testing effect in the present study, it is closely related to one of the most important methodological differences between testing effect studies and other more classic episodic memory paradigms. The main paradigms used to examine episodic long-term memory during development, which have demonstrated improvements in binding operations, relational semantic processing and mnemonic control operations, did not involve long retention intervals (e.g., 1 or 2 days) and did not comprise overnight sleep periods. To better understand developmental differences related to the ability to benefit from testing, future research should focus on analyzing differences in sleep efficiency in developmental samples and adults, as well as how sleep cycles and particularly spindle pattern changes during childhood impact long-term memory retention as a function of how information is encoded. Moreover, it would be great if future research can replicate these findings across diverse cultural and educational settings, using alternative materials, and broaden the range of cognitive covariates examined (e.g., attentional control, further executive functions).

Results from the present study could have an impact in terms of implementing the testing effect in applied settings. According to our data, children can further benefit from testing around age 10. However, it is worth mentioning that the younger-aged group did not show any negative effects from the repeated retrieval relative to the repeated study encoding strategy. And other studies have shown the testing effect can be present in children as young as 3–5 years old (Fritz et al., 2007). This leads us to suggest that according to our data repeated retrieval encoding could be implemented as early as 1st grade in elementary school, since it has been shown to benefit other aspects of learning such as anxiety control (Agarwal et al., 2014). Training in encoding through testing from the early stages of school may also lead to an improved capacity to benefit from testing in the future.

## Data Availability

The datasets presented in this study can be found in online repositories. The names of the repository/repositories and accession number(s) can be found below: https://osf.io/j5y29/.
